# The effect of surfactant on clinical outcome of patients with COVID-19 under mechanical ventilation: A structured summary of a study protocol for a randomised controlled trial

**DOI:** 10.1186/s13063-020-04815-z

**Published:** 2020-11-11

**Authors:** Ali Dabbagh, Samira Rajaei, Mehdi Ghahremani, Mohammad Fathi, Nilofar Massoudi, Sasan Tavana, Kamal Fani, Navid Nooraee, Nasser Malekpour Alamdari, Sara Besharat, Arash Najafi Abrandabadi, Ali Pirsalehi, Mohammad Ali Khabiri Khatiri

**Affiliations:** 1 Cardiac Anesthesiology Department, Anesthesiology Research Center, School of Medicine, Shahid Beheshti University of Medical Sciences, Tehran, Iran; 2 Immunology Department, School of Medicine, Tehran University of Medical Sciences, Tehran, Iran; 3 Fellowship of Critical Care Medicine, Anesthesiology Department, School of Medicine, Shahid Beheshti University of Medical Sciences, Tehran, Iran; 4 Anesthesiology Department, School of Medicine, Shahid Beheshti University of Medical Sciences, Tehran, Iran; 5 Internal Medicine Department, School of Medicine, Shahid Beheshti University of Medical Sciences, Tehran, Iran; 6 Fellowship of Cardiac Anesthesia, Anesthesiology Department, School of Medicine, Shahid Beheshti University of Medical Sciences, Tehran, Iran; 7 Department of Surgery, School of Medicine, Shahid Beheshti University of Medical Sciences, Tehran, Iran; 8 Department of Radiology, School of Medicine, Shahid Beheshti University of Medical Sciences, Tehran, Iran

**Keywords:** COVID-19, SARS-COV-2, surfactant, ICU, acute respiratory distress syndrome, ventilator, mechanical ventilation; mortality; hospital length of stay; hospital discharge rate Randomised controlled trial, protocol

## Abstract

**Objectives:**

Assessing the effect of surfactant on clinical outcome in patients with COVID-19 under mechanical ventilation

**Trial design:**

**Single centre**, two arm, parallel group (1:1 allocation ratio), randomised superiority trial with blinded care and outcome assessment.

**Participants:**

Inclusion criteria: Adult COVID-19 patients admitted to the ICU in Modarres hospital, Tehran, Iran (age range of 18 to 99 years) with moderate to severe ARDS (based on definition of P/F ratio) requiring auxiliary respiratory devices (either intubation or face mask).

Exclusion criteria:

● Existence of a major underlying pulmonary disease in addition to COVID-19

● Underlying congenital heart disease

● Patients needing extracorporeal membrane oxygenation (ECMO)

● ARDS primarily due to any other reason rather than COVID-19

● The primary source of pulmonary involvement was bacterial pneumonia or any other etiology except for COVID-10 induced lung involvement

● Those who refused to continue the study (either the patient or their family)

● any patient had any sign of healing before entering the study leading to discharge from ICU in less than 12 hours

**Intervention and comparator:**

In the intervention group, the dose of the drug is a vial containing 4 ml, equivalent to 100 mg, which is prescribed for an adult weighing about 70 kg each time, and if the patient's weight is much lower or higher, it will be adjusted accordingly. Surfactant is prescribed inside the trachea in two doses, starting on the day of intubation with a second dose 6 hours later. The control group will receive the same volume of normal saline, based on weight, administered into the trachea with the same time schedule.

**Main outcomes:**

30 days mortality; patient mortality during stay in ICU up to 30 days; ICU length of stay up to 30 days; Time under mechanical ventilation up to 30 days.

**Randomisation:**

After the participant enters the study, i.e. after the qualification of the patients in the trial is confirmed and their informed written consent is taken, we will use a simple randomisation method using a table of random numbers. In order to hide the random allocation process, a central randomisation approach will be used and the random sequence will be at the disposal of one of the researchers, excluding the principal investigator.

**Blinding (masking):**

Participants, healthcare providers and the principal investigator assessing the outcomes will all be blinded to the group assignment.

**Numbers to be randomised (sample size):**

A total of 60 participants will be randomised in a 1:1 allocation ratio (30 patients allocated to the intervention group and 30 patients allocated to the control group).

**Trial Status:**

The protocol is Version 1.0, May 31, 2020. Recruitment began July 30, 2020, and is anticipated to be completed by October 30, 2020.

**Trial registration:**

IRCT registration number: IRCT20091201002804N12

Registration date: 1st June 2020, 1399/03/12

**Full protocol:**

The full protocol is attached as an additional file, accessible from the Trials website (Additional file 1). In the interest in expediting dissemination of this material, the familiar formatting has been eliminated; this Letter serves as a summary of the key elements of the full protocol.

**Supplementary Information:**

**Supplementary information** accompanies this paper at 10.1186/s13063-020-04815-z.

## Supplementary Information

**Additional file 1.**

## Data Availability

The corresponding author has access to the final dataset of the trial, and the data will be available on reasonable request (Contact: alidabbagh@yahoo.com).

