# Supplementary material for: The effect of surfactant on clinical outcome of patients with COVID-19 under mechanical ventilation: A structured summary of a study protocol for a randomised controlled trial
Source: Trials. 2020 Nov 11;21:919. doi: 10.1186/s13063-020-04815-z (PMC7656195; doi:10.1186/s13063-020-04815-z)
Supplement: Supplementary file 1 — Additional file 1. [file 13063_2020_4815_MOESM1_ESM.pdf]

The effect of surfactant on clinical outcome of patients with COVID-19 under mechanical ventilation

More options

Protocol summary

Study aim

Assessing the effect of surfactant on clinical outcome in patients with Covid-19 under mechanical ventilation

Design

Two arm parallel group randomized trial with blinded care and outcome assessment

Settings and conduct

COVID-19 patients under mechanical ventilation would enter the study; one group would receive the standard treatment added with placebo and the other group would receive standard care plus intra-tracheal surfactant

Participants/Inclusion and exclusion criteria

Inclusion criteria: If the patient is intubated and under mechanical ventilation with SpO2<85% If the patient has confirmed COVID-19 Exclusion criteria: Existence of a major underlying pulmonary disease in addition to COVID-19 Underlying congenital heart disease

Intervention groups

In the intervention group, based on the dose announced in the study protocol, surfactant is prescribed inside the trachea in two doses at a distance of 6 hours, and at the same time, the dependent variables of the study are measured. At the same time, in the control group, the same volume of normal saline is administered in the trachea within the same time schedule

Main outcome variables

patient mortality; ICU length of stay; Time to be under mechanical ventilation

General information

Reason for update

Acronym

IRCT registration information

IRCT registration number: **IRCT20091201002804N12**
Registration date: **2020-06-01, 1399/03/12**
Registration timing: **registered_while_recruiting**

Last update: **2020-06-01, 1399/03/12**
Update count: **0**

Registration date

2020-06-01, 1399/03/12

Registrant information

Name
Ali Dabbagh
Name of organization / entity
Country
Iran (Islamic Republic of)
Phone
+98 21 2243 2572
Email address
alidabbagh@sbmu.ac.ir

Recruitment status

Recruitment complete

Funding source

Expected recruitment start date

2020-04-20, 1399/02/01

Expected recruitment end date

2020-06-21, 1399/04/01

Actual recruitment start date

empty

Actual recruitment end date

empty

Trial completion date

empty

Scientific title

The effect of surfactant on clinical outcome of patients with COVID-19 under mechanical ventilation

Public title

The effect of surfactant on clinical outcome of patients with COVID-19

Purpose

Treatment

Inclusion/Exclusion criteria

Inclusion criteria:
if the patient is intubated and under mechanical ventilation with SpO2<85% If the patient has confirmed COVID-19
Exclusion criteria:
Coexisting underlying pulmonary disease except for COVID-19 Underlying congenital hear disease

Age

From **18 years** old to **99 years** old

Gender

Both

Phase

3

Groups that have been masked

- Participant
- Care provider
- Investigator

Sample size

Target sample size: **60**

Randomization (investigator's opinion)

Randomized

Randomization description

After the participant enters the study, i.e. after the qualification of the patients in the trial is confirmed and their informed written consent is taken, simple randomization will be done as follows: 1- Table of random numbers will be used for creation of coincidence of random allocation. 2- In order to hide the random allocation process, the central randomization approach will be used, while the random sequence would be at the disposal of one of the researchers except for the principal investigator.

Blinding (investigator's opinion)

Triple blinded

Blinding description

participants after entering the study would not know whether they are in the drug or placebo group healthcare providers (Physicians and nurses) would administer the prepared vial including drug or placebo while they do not know its content; the vial would be assimilated; regardless of drug or placebo principle investigator does not know whether the patient belongs to the drug group or the placebo group since the patients have been randomized

Placebo

Not used

Assignment

Factorial

Other design features

COVID-19 treatment study

Secondary Ids

empty

Ethics committees

1

Ethics committee

Name of ethics committee
Shahid Beheshti University of Medical Sciences

Street address
Deputy for Research and Technology, Shahid Beheshti University of Medical Sciences, Velenjak

City
Tehran

Province
Tehran

Postal code
1985717443

Approval date

2020-03-28, 1399/01/09

Ethics committee reference number

IR.SBMU.RETECH.REC.1399.016

Health conditions studied

1

Description of health condition studied

Mortality of Patients from COVID-19

ICD-10 code

U07.1

ICD-10 code description

COVID-19

Primary outcomes

1

Description

time for mechanical ventilation

Timepoint

throughout the study, the time that patient has stayed under mechanical ventilation

Method of measurement

clinical records

Secondary outcomes

1

Description

ICU mortality rate

Timepoint

throughout the study in the ICU ward

Method of measurement

clinical records

Intervention groups

1

Description

Intervention group: Intra-tracheal surfactant in COVID-19 patients who are under mechanical ventilation, which includes the administration of a standard dose of surfactant inside the airway of the patient with COVID-19 diagnosis, which is administered immediately on the first day of intubation and in two doses at intervals within 6 hours. The dose of the drug is a vial containing 4 ml, equivalent to 100 mg, which is prescribed for an adult weighing about 70 kg each time, and if the patient's weight is higher, it will be adjusted accordingly. The drug is from the brand Beraksurf® and is supplied by Tekzima.

Category

Treatment - Drugs

2

Description

Control group: all the treatment protocols including standard of care is the same as the treatment group; except for the intrathecal administration of surfactant. An equivalent volume of normal saline is used as placebo

Category

Placebo

Recruitment centers

1

Recruitment center

Name of recruitment center
Modarres hospital

Full name of responsible person
Ali Dabbagh

Street address
Sa'adat Abad

City
Tehran

Province
Tehran

Postal code
1998738341

Phone
+98 21 2207 4100

Fax
+98 21 2207 4101

Email
alidabbagh@yahoo.com

Sponsors / Funding sources

1

Sponsor

Name of organization / entity
Shahid Beheshti University of Medical Sciences

Full name of responsible person

Afshin Zarghi

Street address
Velenjak, Chamran Exp Way

City
Tehran

Province
Tehran

Postal code
1983535511

Phone
+98 21 2387 2202

Fax
+98 21 2387 2202

Email
info@sbmu.ac.ir

**Grant name**	
**Grant code / Reference number**	
**Is the source of funding the same sponsor organization/entity?**	No
**Title of funding source**	Shahid Beheshti University of Medical Sciences
**Proportion provided by this source**	100
**Public or private sector**	Public
**Domestic or foreign origin**	Domestic
**Category of foreign source of funding**	*empty*
**Country of origin**	
**Type of organization providing the funding**	

Academic

Person responsible for general inquiries

Contact

Name of organization / entity
Shahid Beheshti University of Medical Sciences

Full name of responsible person
Ali Dabbagh

Position
Professor

Latest degree
Subspecialist

Other areas of specialty/work
Anesthesiology

Street address
Sa'adat Abad

City
Tehran

Province
Tehran

Postal code
1998738341

Phone
+98 21 2387 2202

Email
alidabbagh@yahoo.com

Person responsible for scientific inquiries

Contact

Name of organization / entity
Shahid Beheshti University of Medical Sciences

Full name of responsible person
Ali Dabbagh

Position
Professor

Latest degree
Subspecialist
Other areas of specialty/work
Anesthesiology
Street address
Sa'adat Abad
City
Tehran
Province
Tehran
Postal code
1998738341
Phone
+98 21 2207 4101
Email
alidabbagh@yahoo.com

Person responsible for updating data

Contact

Name of organization / entity
Shahid Beheshti University of Medical Sciences
Full name of responsible person
Ali Dabbagh
Position
Professor
Latest degree
Subspecialist
Other areas of specialty/work
Anesthesiology
Street address
Sa'dat Abad
City
Tehran
Province
Tehran
Postal code
1998738341
Phone
+98 21 2207 4101

Email
alidabbagh@yahoo.com

Sharing plan

**Deidentified Individual Participant Data Set (IPD)**	Yes - There is a plan to make this available
**Study Protocol**	Yes - There is a plan to make this available
**Statistical Analysis Plan**	Yes - There is a plan to make this available
**Informed Consent Form**	Yes - There is a plan to make this available
**Clinical Study Report**	Yes - There is a plan to make this available
**Analytic Code**	Yes - There is a plan to make this available
**Data Dictionary**	Yes - There is a plan to make this available
**Title and more details about the data/document**	all collected deidentified IPD
**When the data will become available and for how long**	starting in January 2022
**To whom data/document is available**	the data would be available for people working in academic institutions and people working in businesses
**Under which criteria data/document could be used**	

	by formal permission form PI
**From where data/document is obtainable**	Address of the Principal Investigator
**What processes are involved for a request to access data/document**	Formal application confirmed by the institution
**Comments**	

- Home (/)
- About IRCT (/)
- Contact us (/)
- Help (/)

Tel:
Working hours:
8:00 - 15:30 Tehran time
11:30 - 19:00 GMT

0098 21 8670 5503

During COVID-19 Epidemic at working times:
0098 936 770 7834

Fax:
0098 21 8670 5503

Email:
admin@irct.ir (mailto:admin@irct.ir)

Directly contacting the manager:
0098 912 778 2686

Address:
IRCT administration team,
Central Library Building, Iran University Campus,
Hemmat freeway, next to Milad tower,
Tehran, 14496-14535
Iran

Copyright © IRCT 2008-2020. All Rights Reserved.
